# AMD1-mediated polyamine metabolism governs tubular repair fate by restraining senescence after kidney injury

**DOI:** 10.1080/0886022X.2026.2680375

**Published:** 2026-06-14

**Authors:** Baiwei Mao, Zhihuang Zheng, Wenxin Fu, Guozhe Cheng, Lijun Wang, Jinfang Bao, Xiaohua Liu, Hongbin Zhan, Miao Pan, Jun Liu

**Affiliations:** ^a^ Department of Nephrology, Shanghai General Hospital, Shanghai Jiaotong University School of Medicine, Shanghai, China; ^b^ Laboratory of Nephropathy, Translational Medicine Center, Shanghai General Hospital, Shanghai Jiaotong University School of Medicine, Shanghai, China; ^c^ Institute of Translational Medicine, Shanghai General Hospital, Shanghai Jiaotong University School of Medicine, Shanghai, China; ^d^ Department of Nephrology, Ningde Municipal Hospital of Ningde Normal University, Ningde, China; ^e^ Department of Nephrology, Shanghai First People’s Hospital Ningde Hospital, Ningde, China

**Keywords:** AKI-to-CKD, polyamine, spermidine, tubular senescence, p53/p21

## Abstract

Failure of adaptive repair after acute kidney injury (AKI) drives the transition to chronic kidney disease (CKD), yet the metabolic checkpoints governing tubular fate remain incompletely defined. Here, we investigated whether the polyamine biosynthetic enzyme S-adenosylmethionine decarboxylase 1 (AMD1) regulates tubular senescence and repair outcomes after AKI and elucidated the underlying mechanism. AMD1 dynamics were examined in an ischemia-reperfusion injury model using male C57BL/6J mice by immunofluorescence. AAV-mediated Ksp promoter-driven tubule-specific *Amd1* conditional knockdown male mice (*Amd1* ^cKD^) were used to assess renal injury, cell-cycle status, senescence, and remodeling, and exogenous spermidine was administered for rescue. DNA damage signaling and p53/p21 activation were evaluated by immunostaining, Western blotting, and EdU incorporation assays. AMD1 was predominantly expressed in the tubular epithelium, with prominent dynamic induction in proximal tubules early after IRI, but declined to baseline levels during the late phase, representing a relative metabolic insufficiency that correlated inversely with fibrosis. Compared with wild-type controls, *Amd1* ^cKD^ mice exhibited aggravated tubular injury, an over two-fold increase in SA-β-gal-positive areas, elevated p21, and reduced Ki67+ proliferation. Conversely, spermidine supplementation improved renal function, reduced fibrosis by 75.3%, and decreased senescent regions by 74%. Mechanistically, AMD1 deficiency increased γH2AX-marked DNA damage and activated the p53/p21 checkpoint, whereas spermidine attenuated this response and restored DNA synthesis capacity. Collectively, tubular AMD1 acts as a metabolic checkpoint that preserves polyamine homeostasis to restrain p53/p21-dependent senescence, promote adaptive repair after AKI, and spermidine supplementation represents a potential strategy to mitigate maladaptive AKI-to-CKD progression.

## 1. Introduction

Clinically, acute kidney injury (AKI) can result in complete structural and functional recovery if adaptive repair mechanisms are successfully engaged. Conversely, severe and persistent injury frequently triggers maladaptive repair, driving the pathogenic transition from AKI to chronic kidney disease (CKD) [1–3]. This transition is driven by a complex interplay of molecular mechanisms, encompassing sustained cell-cycle arrest, severe inflammatory responses, DNA damage, mitochondrial dysfunction, and epigenetic reprogramming. Ultimately, the trajectory of this transition hinges on the fate of injured tubular epithelial cells. While successful repair requires cellular regeneration, maladaptive repair is marked by irreversible cell-cycle arrest and the induction of tubular senescence. Through the continuous secretion of a senescence-associated secretory phenotype (SASP), these senescent cells actively drive interstitial remodeling and accelerate renal fibrosis [4].

Metabolic reprogramming has emerged as a key regulator of tubular fate. While prior studies have largely focused on lipid dysregulation, mitochondrial dysfunction and glycolytic rewiring [4,5], the contribution of amino acid-derived metabolites remains underexplored. Polyamines are cationic metabolites essential for chromatin organization, DNA repair, and cell-cycle progression, and their tissue levels decline markedly under severe cellular stress and senescence [6–8]. Despite growing links between polyamine metabolism and cell senescence [9], how polyamine homeostasis shapes tubular repair trajectories during AKI-to-CKD progression is still poorly defined.

S-adenosylmethionine decarboxylase 1 (AMD1) is a rate-limiting enzyme in polyamine biosynthesis and a metabolic node connecting polyamine production with one-carbon metabolism [7,10]. Previous work has emphasized AMD1-mediated perturbation of methyl-donor balance, specifically the ratio of *S*-adenosylmethionine (SAM) to decarboxylated SAM (dcSAM), and the subsequent epigenetic instability. However, this methylation-centered view may overlook the essential role of AMD1 in supplying higher bioactive polyamines, such as spermidine and spermine, that directly support genome integrity and proliferative competence [10].

Here, we integrate public transcriptomic mining with *in vivo* and *in vitro* validation to identify AMD1 as a renal tubule-enriched enzyme that becomes markedly upregulated in the early phase but fails to sustain this induction, returning to baseline levels during the late phase following ischemic injury. Using AAV-mediated Ksp promoter-driven tubule-specific *Amd1* conditional knockdown mice, we show that tubular AMD1 deficiency exacerbates renal dysfunction and fibrotic remodeling by promoting DNA damage accumulation and activating the p53/p21 checkpoint, thereby enforcing tubular senescence and senescence-associated secretory phenotype (SASP) programs. Importantly, exogenous spermidine supplementation suppresses p53/p21 activation, restores DNA synthesis and proliferation, and markedly improves functional and structural repair. Together, these findings establish AMD1 as a metabolic checkpoint controlling post-injury tubular fate and highlight polyamine restoration as a potential strategy to mitigate maladaptive AKI-to-CKD progression.

## 2. Materials and methods

### 2.1. Bioinformatics analysis and phenotypic module scores

To quantify the activity of specific biological programs at the individual sample level, two distinct computational approaches were employed based on the nature of the gene sets.

#### 2.1.1. Single-sample Gene Set Enrichment Analysis (ssGSEA)

Bulk RNA-seq data from the GSE165876 dataset were log2-transformed for normalization. Differential gene expression analysis between the IRI and Sham groups was performed using the limma R package. Multiple testing correction was applied using the Benjamini-Hochberg false discovery rate (FDR) method. Genes meeting the strict criteria of an adjusted p<0.05 and $|{\text{log}}_{2}\text{FC}|>0.5$ were defined as differentially expressed genes (DEGs).

**Figure 1.**
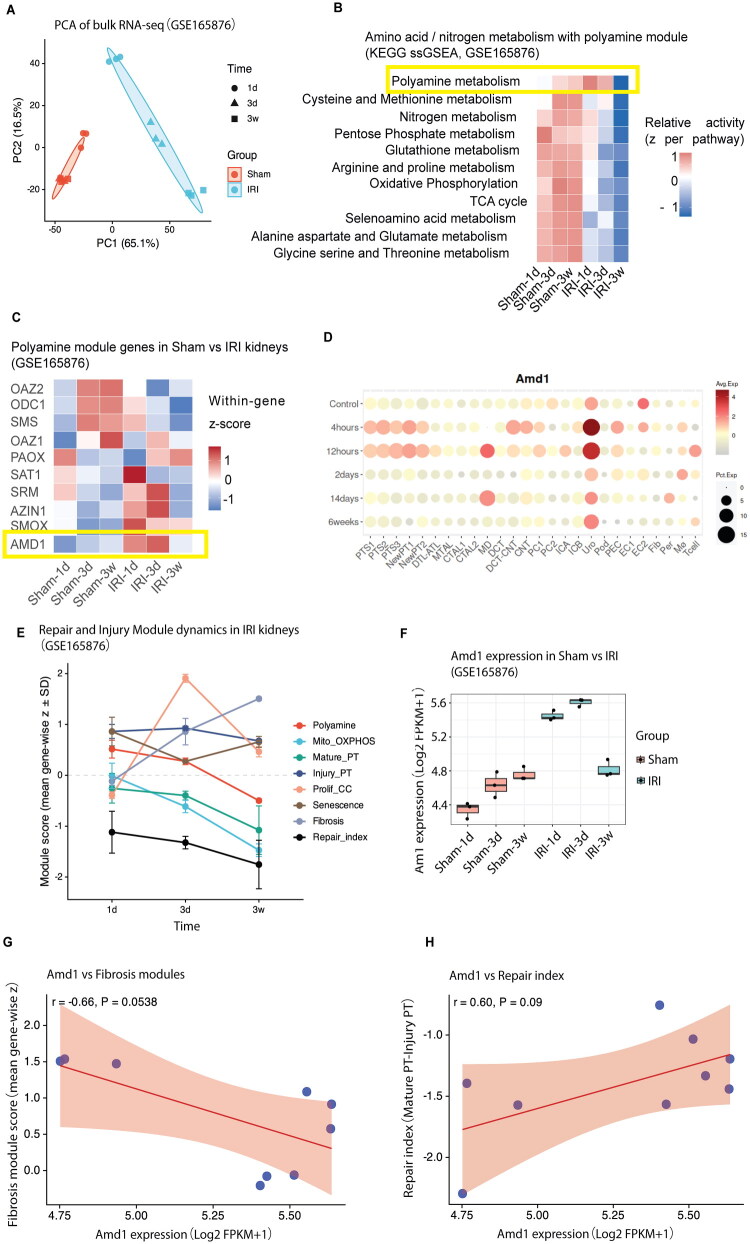
Downregulation of AMD1-mediated polyamine metabolism is closely associated with maladaptive repair outcomes after acute kidney injury (AKI). (A, B) Principal component analysis (PCA) of the public bulk RNA-seq dataset GSE165876 (A) and single-sample Gene Set Enrichment Analysis (ssGSEA)-based scoring of amino acid/nitrogen metabolic KEGG pathways (B). The heatmap illustrates that the polyamine metabolism module exhibits a distinct biphasic pattern, characterized by compensatory activation followed by significant suppression during the late phase (3 weeks) after ischemia-reperfusion injury (IRI). (C) Heatmap profiling the relative expression (within-gene Z-score) of core genes in the polyamine metabolism module, highlighting AMD1 as a prominently downregulated node during late-stage IRI. (D) Dot plot visualization of *Amd1* single-cell RNA-seq (scRNA-seq) expression dynamics across distinct renal cell populations post-IRI (derived from Humphreys Lab Kidney Biobank) [11]. *Amd1* is broadly expressed across the tubular epithelium (including proximal tubules [PTs], principal cells [PCs], and intercalated cells [ICs]). Notably, within the PT populations (PTS1-3 and NewPT), *Amd1* exhibits transient, robust induction during the early acute phase (4–12 h) followed by a return to baseline or decline during the late phase (14 days to 6 weeks). The dot size represents the percentage of cells expressing the gene, and the color scale indicates the average expression level. (E, F)Temporal dynamics of phenotypic module scores related to injury, repair, and senescence across IRI progression (E), alongside quantitative bulk expression analysis of *Amd1* (Log2 FPKM + 1) at corresponding time points (F). Data are presented as mean ± SEM. (G, H) Correlation analyses demonstrating that *Amd1* expression is negatively correlated with the fibrosis module score (G) and positively associated with the proximal tubule repair index (H). The shaded areas represent the 95% confidence intervals. Statistical significance and correlation coefficients (r) were determined using Pearson correlation analysis.

For the evaluation of canonical metabolic pathways (Figure 1B), we utilized the single-sample Gene Set Enrichment Analysis (ssGSEA) algorithm implemented in the GSVA R package. Reference gene sets for standard energy and amino acid metabolism were obtained from the KEGG database *via* the msigdbr R package.

To quantitatively evaluate polyamine flux, a specific ‘polyamine score’ was defined using a targeted module comprising 10 essential genes (*Oaz2, Odc1, Sms, Oaz1, Paox, Sat1, Srm, Azin1, Smox*, and *Amd1*) [6,10,32]. Raw ssGSEA enrichment scores were calculated based on the absolute expression rank of these predefined genes within each sample’s total transcriptome. Equal weighting was applied to all genes within the module. To facilitate visualization of relative activity changes across time points, these raw scores were subsequently normalized across all samples using gene-wise Z-score scaling (mean = 0, standard deviation = 1).

#### 2.1.2. Calculation of cellular phenotypic module scores

To evaluate specific cellular phenotypic shifts, namely adaptive tubular repair and fibrogenesis (Figure 1G,H), module scores were computed using a Z-score arithmetic methodology. The raw FPKM values were log_2_-transformed using the formula ${\text{log}}_{2}\left(\text{FPKM}+1\right)$ to stabilize the variance. Gene-wise Z-score normalization was then applied across all samples, scaling the expression of each target gene to a mean of 0 and a standard deviation of 1. A basic module score for a given gene set was defined as the unweighted arithmetic mean of the Z-scores of its constituent genes.

The fibrosis module score was calculated based on gold-standard markers of myofibroblast activation and extracellular matrix deposition (*Acta2, Col1a1, Col3a1, Fn1,* and *Postn* [33]). The repair index was designed to quantify the net phenotypic shift between the mature and injured proximal tubule (PT) states. This index was calculated by subtracting the injury module score (based on markers of maladaptive repair and dedifferentiation: *Sox9, Havcr1, Vim, Krt8,* and *Cd44* [11,34]) from the mature module score (based on markers encompassing the core functional and metabolic features of a differentiated PT: *Slc27a2, Slc22a6, Slc3a1, Hnf4a,* and *Pck1* [11,35]). No artificial weighting factors were applied to individual genes in these calculations.

#### 2.1.3. Single-cell RNA sequencing data query

To investigate the cellular distribution and temporal expression profile of *Amd1* in the kidney following ischemia-reperfusion injury (IRI), we performed a bioinformatic query using the Humphreys Lab Kidney Single Cell Database (http://humphreyslab.com/SingleCell/) [11], which includes scRNA-seq profiles from mouse kidneys at multiple time points post-IRI (Sham, 4 h, 12 h, 2 d, 14 d, and 6 weeks). *Amd1* expression levels were analyzed across distinct renal cell clusters, including proximal tubule (PT) segments, distal tubules, and collecting ducts. Data visualization and cluster-specific expression analysis were conducted using the platform’s integrated analysis tools.

### 2.2. Animals and ethics

8-week-old male C57BL/6J mice weighing 23 to 25 g were purchased from Shanghai SLAC Laboratory Animal Co., Ltd. and housed under specific pathogen-free (SPF) conditions at the Experimental Animal Center of Shanghai General Hospital, Shanghai Jiao Tong University School of Medicine. Mice were housed in individually ventilated cages (IVC) with standard bedding, maintained on a 12-h light/dark cycle at 22 ± 2 °C and 40%–60% humidity, and provided with *ad libitum* access to standard rodent chow and sterile water. Environmental enrichment, such as nesting material, was provided in all cages. All procedures were approved by the Institutional Animal Care and Use Committee (IACUC) of Shanghai General Hospital (Approval No. 2021AW016) and were conducted in accordance with relevant guidelines for the humane use and care of laboratory animals.

### 2.3. Anesthesia and euthanasia

For anesthesia, mice were administered a single intraperitoneal (IP) injection of 1% sodium pentobarbital (Sigma-Aldrich, St. Louis, MO, USA, Cat# Y0002194, 40 mg/kg) at a dose of 40 mg/kg body weight, which is approximately 0.1 mL for a 25 g mouse. To prevent hypothermia during anesthesia, mice were placed on a thermostatically controlled heating pad at 37 °C until they fully recovered. At the experimental endpoint, euthanasia was performed by an overdose of sodium pentobarbital (150 mg/kg, IP) followed by cervical dislocation, ensuring minimal pain and distress in compliance with the American Veterinary Medical Association (AVMA) Guidelines for the Euthanasia of Animals.

### 2.4. Reagents

Detailed information regarding all primary antibodies, assay kits, and other key reagents used in this study, including their commercial sources, catalog numbers, and specific amounts or dilutions used, is provided in Supplementary Table S3.

### 2.5. In situ renal delivery of AAV and generation of tubule-targeted Amd1 ^cKD^ model

To achieve robust and segment-specific suppression of *Amd1* expression in renal tubular epithelial cells *in vivo*, we utilized an adeno-associated virus serotype 9 (AAV9) vector system. The vector was engineered with a Ksp-cadherin (Cadherin-16) promoter to restrict the expression of the downstream shRNA exclusively to the tubular compartment, sparing the glomeruli and interstitium.

To maximize knockdown efficiency and minimize potential off-target effects, a pooled shRNA strategy was employed. The AAV9-KSP-shAmd1 viral cocktail (Hanbio Biotechnology, Shanghai, China) contained a mixture of three distinct shRNAs targeting different regions of the mouse *Amd1* transcript. A corresponding vector expressing a validated scrambled non-targeting sequence was used as a control (AAV9-KSP-shCtrl). The specific target sequences are listed in Supplementary Table S1.

Briefly, 2 weeks prior to AKI induction, mice were anesthetized, and bilateral renal pelvis microinjections were performed using a microsyringe. Each kidney received 50 μL of the viral suspension (2 × 10^12^vg/mL). The 31 G needle was retained in place for 1 min post-injection to facilitate optimal viral dispersion. Mice were subsequently returned to standard housing. The segment-specific knockdown efficiency was independently validated *via* Western blotting and immunofluorescence (IF). Throughout this manuscript, this tubule-targeted knockdown model is referred to as the *Amd1* ^cKD^ model.

### 2.6. Experimental design and renal ischemia-reperfusion injury model

Renal ischemia-reperfusion injury (IRI) was used to induce acute kidney injury (AKI) *in vivo*; herein, this IRI-induced model is collectively referred to as AKI throughout the manuscript. Mice were randomly assigned to four groups (n = 5 per group): WT-sham (WT + AAV-shCtrl + sham surgery), WT-AKI (WT + AAV-shCtrl + AKI), Amd1 cKD-AKI (WT + AAV-shAmd1 + AKI), and Amd1 cKD + Spd-AKI (WT + AAV-shAmd1 + Spd + AKI).

To minimize potential confounders, the order of IRI procedures and subsequent measurements was randomized across experimental groups, and cages were distributed randomly on the holding racks. Animals were included in the study based on *a priori* criteria of age (8 weeks), sex (male), and body weight (23–25 g). No animals or data points were excluded from the analysis. In this study, a single animal was considered as the experimental unit for all analyses.

AKI was induced by bilateral renal pedicle clamping for 25 min followed by reperfusion. Body temperature was maintained at 37 °C throughout surgery. Sham-operated mice underwent identical procedures except for vascular clamping. Mice were euthanized on day 14 after AKI surgery; serum was collected, and kidneys were harvested. Kidney tissues were transversely sectioned and either fixed in 4% paraformaldehyde or snap-frozen and stored at −80 °C for subsequent analyses.

Following surgery, animals were monitored daily for signs of pain, distress, and general health, including body weight, posture, and mobility. Humane endpoints were established *a priori*: any animal exhibiting signs of severe distress, such as >20% body weight loss, prolonged lethargy, or hunched posture, would be euthanized immediately. In this study, no unexpected adverse events or spontaneous mortality occurred prior to the planned experimental endpoints. A formal study protocol was prepared internally before the commencement of the study; however, it was not registered in a public repository.

### 2.7. In vivo spermidine administration

Spermidine was dissolved in sterile normal saline. Mice in the Spd group received Spd (MedChemExpress, Monmouth Junction, NJ, USA, Cat# HY-B1776) by oral gavage at a dose of 10 mg/kg per day, starting 7 days prior to the ischemia reperfusion injury (IRI) procedure and continuing until 14 days after the surgery. Control animals received an equal volume of normal saline as a vehicle following the same administration schedule.

### 2.8. Histopathology and tubular injury/fibrosis scoring

Kidney sections were stained with hematoxylin and eosin (H&E) for histological assessment and Masson’s trichrome staining for evaluation of fibrosis. For each sample, 20 random fields were captured, and tubular injury was scored based on the percentage of damaged tubules (injury area proportion). Fibrotic area was quantified from Masson-stained sections using ImageJ.

### 2.9. Serum biochemistry

Peripheral blood was collected and centrifuged at 3000 rpm for 15 min to obtain serum. Scr and BUN levels were determined according to the manufacturer’s instructions (Nanjing Jiancheng) using the sarcosine oxidase method for Scr and the diacetyl monoxime method for BUN. Absorbance was measured using a microplate reader, and concentrations were calculated from standard curves.

### 2.10. Immunohistochemistry

Paraffin-embedded kidney sections were deparaffinized, rehydrated, subjected to antigen retrieval, and blocked. Sections were incubated overnight at 4 °C with primary antibodies against AQP1, Ki67, α-SMA, p21, or AMD1 (dilutions according to manufacturers’ instructions; alternatively, 1:200-1:400). After incubation with horseradish peroxidase (HRP)-conjugated secondary antibodies, immunoreactivity was visualized with 3,3′-diaminobenzidine (DAB). Positive staining was quantified as the percentage of positive area in 20 random fields per section using ImageJ.

### 2.11. Multiplex Tyramide Signal Amplification (TSA) immunofluorescence

To precisely evaluate protein co-localization and address the severe disease-specific background (e.g., autofluorescence from tubular casts) associated with the ischemic AKI model, an optimized multiplex immunofluorescence protocol was performed using the Tyramide Signal Amplification (TSA) method.

Kidney sections were deparaffinized, rehydrated, and subjected to heat-induced antigen retrieval. To eliminate nonspecific tyramide deposition, sections were strictly treated with 3% hydrogen peroxide to quench endogenous peroxidases, followed by an intensive blocking step using normal serum and 1% BSA containing 0.3% Triton X-100. For multiplex staining, the sections underwent iterative cycles of staining. In each cycle, tissues were incubated with the specific primary antibody, followed by an HRP-conjugated secondary antibody. The fluorescent signal was subsequently generated by the covalent deposition of tyramide fluorophores (TSA-520, TSA-570, or TSA-690). Following each TSA deposition, sections were subjected to microwave heating to strip the previously bound primary and secondary antibodies before initiating the next staining cycle for the subsequent target. Nuclei were counterstained with DAPI, and slides were mounted using an anti-fade mounting medium. Images were acquired using a fluorescence microscope.

### 2.12. Senescence-Associated β-galactosidase (SA-β-gal) staining

Frozen kidney sections (8–10 μm) were fixed and incubated with SA-β-Gal staining solution (pH 6.0) at 37 °C (no CO_2_) overnight. Images were captured under a light microscope, and the SA-β-Gal-positive area was quantified as a percentage of the total tissue area using ImageJ.

### 2.13. RT-qPCR

Total RNA was extracted from renal cortex tissue and reverse-transcribed into cDNA. Quantitative PCR was performed using SYBR Green chemistry to quantify mRNA levels of SASP-associated genes, including *Cxcl1, Il1b, Il6, Cdkn1a*, and *Cdkn2a*. Gapdh was used as the internal control, and relative expression was calculated using the 2^−ΔΔCt^ method. The specific primer sequences used in this study are listed in Supplementary Table S4.

### 2.14. Cell culture and siRNA transfection

Human proximal tubular epithelial cells (HK-2; Procell, Wuhan, China) were cultured in DMEM/F12 medium supplemented with 10% fetal bovine serum (FBS). Cells in logarithmic growth phase were seeded and transfected with SiAMD1 or negative control SiRNA (SiNC) using Lipofectamine 3000 according to the manufacturer’s protocol. After 6 h, the medium was replaced. For rescue experiments, Spd was added to the culture medium at 10 μM and incubated for 24–48 h. The SiRNA sequences were listed in Supplementary Table S2.

### 2.15. EdU incorporation assay

HK-2 cells cultured on coverslips were incubated with 10 μM EdU for 2 h, fixed, permeabilized, and stained using an Apollo reaction cocktail followed by nuclear counterstaining with Hoechst 33342. Five random fields per coverslip were imaged, and the proportion of EdU-positive cells was quantified using ImageJ.

### 2.16. Statistical analysis

Data are presented as mean ± standard error of the mean (SEM). Data normality and homogeneity of variance were verified using Shapiro-Wilk and Brown-Forsythe tests, respectively. Statistical analyses were performed using GraphPad Prism 9.5 (GraphPad Software, San Diego, CA, USA). Comparisons among multiple groups were conducted using one-way ANOVA followed by Tukey’s *post hoc* test. A two-tailed p-value < 0.05 was considered statistically significant. The investigators were blinded to group allocation during data collection and analysis.

## 3. Results

### 3.1. Relative insufficiency of the polyamine enzyme AMD1 is closely associated with impaired repair and activation of senescence programs after IRI

To explore metabolic determinants underlying maladaptive repair during the AKI-to-CKD transition, we interrogated a public bulk RNA-seq dataset (GSE165876) derived from a mouse model of renal ischemia-reperfusion injury (IRI). Principal component analysis (PCA) showed a clear separation between Sham and IRI kidneys across the post-injury time course, indicating robust transcriptional reprogramming during injury and repair (Figure 1A). We then performed pathway-level interrogation using single-sample Gene Set Enrichment Analysis (ssGSEA) based on Kyoto Encyclopedia of Genes and Genomes (KEGG) pathways. This analysis revealed that amino acid and nitrogen metabolism programs, including pathways related to polyamine biosynthesis, exhibited a biphasic pattern, characterized by an early compensatory activation after IRI followed by marked suppression at the late stage (3 weeks) (Figure 1B).

Focusing on the polyamine module, heatmap profiling of core polyamine metabolism genes revealed diverse expression trajectories across the post-injury time course. Notably, *Amd1* exhibited a distinct biphasic pattern, characterized by significant upregulation during the early injury phase (1 to 3 days post-IRI) followed by marked downregulation at the late stage (3 weeks) (Figure 1C). Given its established role as a rate-limiting enzyme in polyamine biosynthesis, we selected *Amd1* as our primary target for further investigation. To resolve the cellular origin of *Amd1* within the kidney, we examined single-cell RNA-seq expression patterns from the Humphreys Lab [11]. Analysis revealed that *Amd1* is broadly expressed across the renal tubular epithelium in the renal cortex. Proximal tubule (PT) cells are the most vulnerable cell type to ischemic injury and act as the primary initiators of the pathogenic cascade. Because the injury, senescence, and maladaptive repair of PT cells serve as the central drivers of the AKI-to-CKD transition [12,13], we specifically focused on the dynamics of *Amd1* regulation within this population. In the PT cells (PTS1-3 and NewPT), *Amd1* expression was transiently induced during the early injury phase (4–12 h post-IRI), but subsequently declined and plateaued at baseline levels during the late phase (14 days to 6 weeks), manifesting a distinct biphasic dynamic (Figure 1D).

We next investigated whether the dynamics of polyamine metabolism correlate with established injury and repair trajectories following IRI. Module-based analyses demonstrated that the polyamine score declined in parallel with increased senescence- and fibrosis-associated signatures, consistent with an inverse relationship between polyamine metabolic capacity and maladaptive repair features (Figure 1E). In line with this, bulk expression analysis confirmed that *Amd1* mRNA levels were significantly reduced in AKI kidneys at later time points compared with early phase after IRI (Figure 1F). Correlation analyses further supported the clinical-pathological relevance of this axis: Amd1 expression negatively correlated with the fibrosis module (Figure 1G), while showing a positive association with the repair index (Figure 1H).

Collectively, these data identify that the return of AMD1 to baseline levels as a characteristic late-stage event after AKI, and link this relative metabolic insufficiency to fibrotic remodeling and senescence-associated maladaptive repair, implicating AMD1 insufficiency as a potential metabolic trigger for senescence and senescence-associated secretory phenotype (SASP) programs during AKI-to-CKD progression.

### 3.2. In situ validation reveals biphasic tubular AMD1 dynamics after AKI and an inverse association with fibrotic remodeling

**Figure 2.**
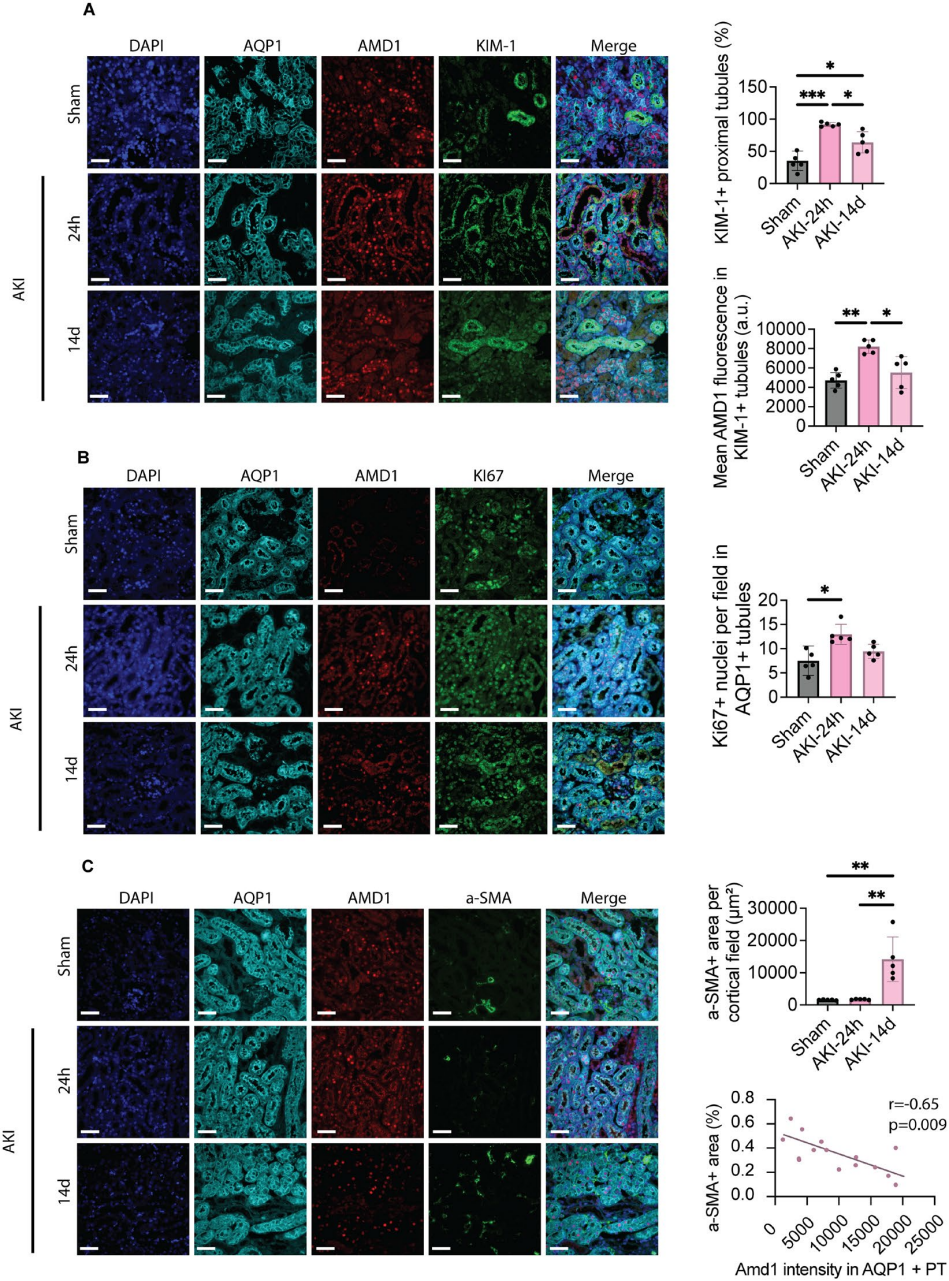
Spatiotemporal dynamics of tubular AMD1 expression and its association with fibrotic remodeling following AKI. (A) Representative multiplex immunofluorescence images and quantification showing the dynamic induction of AMD1 (red) within injured proximal tubules. Tissues were co-stained for the proximal tubule marker AQP1 (cyan), the injury marker KIM-1 (green), and DAPI (blue) at the indicated time points post-IRI. Note the loss of apical membrane polarity and diffuse cytoplasmic redistribution of AQP1, a characteristic pathological hallmark of severe ischemic tubular injury. (B) Representative co-staining and quantification of AMD1 (red) and the proliferation marker Ki67 (green) within AQP1+ proximal tubules (cyan). To strictly exclude nonspecific cytosolic autofluorescence commonly arising from necrotic debris and proteinaceous casts in the ischemic kidney, Ki67+ quantification was exclusively restricted to fluorescent signals perfectly co-localizing with DAPI-stained nuclei. (C) Representative immunofluorescence and correlation analysis demonstrating that the late-phase reduction of AMD1 (red; day 14) is accompanied by the prominent expansion of α-SMA (green), indicating enhanced fibrotic remodeling. Scale bars = 50 μm. *n* = 5 per group. Data are presented as mean ± SEM. Statistical significance among multiple groups (A, B, C bar graphs) was determined using one-way ANOVA followed by Tukey’s *post hoc* test. Correlation analysis (C, scatter plot) was performed using Pearson correlation (*r* = −0.66, *p* = 0.008). *p < 0.05, **p < 0.01, ***p < 0.001.

To validate the bioinformatics-derived AMD1 dynamics at the tissue level, we performed multiplex immunofluorescence staining in a mouse renal IRI induced AKI model to map the spatiotemporal expression of AMD1 within proximal tubules. Under basal conditions (Sham), AMD1 exhibited low-level constitutive staining in AQP1+ proximal tubules (Figure 2A). Strikingly, during the acute injury phase (24 h post-AKI), AMD1 fluorescence intensity was markedly increased in proximal tubules, indicating an early injury-responsive induction (Figure 2A). Co-staining with the injury marker KIM-1 further demonstrated that AMD1 upregulation was preferentially enriched in KIM-1+ damaged tubules, suggesting that AMD1 is activated predominantly within injured epithelial compartments during early post-ischemic stress (Figure 2A, right). High-resolution imaging revealed that AMD1 exhibited a distinct nucleocytoplasmic distribution within tubular epithelial cells, consistent with its biological role in both polyamine synthesis and potential nuclear functions (Figure 2A). However, as the injury course progressed to day 14, AMD1 signal declined relative to the acute phase. Although returning to baseline levels, this represents a relative late-stage AMD1 insufficiency pattern, as the basal expression fails to meet the heightened metabolic demands observed during maladaptive repair (Figure 2A).

We next investigated whether the dynamics of AMD1 expression correlate with epithelial proliferative responses. Co-immunofluorescence for Ki67 showed that tubular proliferation peaked at 24 h, coinciding with the early rise in AMD1 expression, but declined substantially by day 14, when AMD1 levels had fallen (Figure 2B). This temporal coupling supports the notion that AMD1-associated metabolic capacity may be linked to the regenerative competence of proximal tubular cells during repair.

Finally, to connect late-phase AMD1 deficiency with tissue remodeling, we assessed interstitial fibrosis using α-SMA staining. Compared with earlier time points, α-SMA+ area was prominently expanded at day 14 post-AKI, coinciding with reduced AMD1 intensity in proximal tubules (Figure 2C). Quantitative correlation analysis further confirmed that tubular AMD1 intensity was significantly and inversely correlated with α-SMA+ area (Figure 2C), supporting a tight association between declining AMD1 in proximal tubules and the emergence of fibrotic remodeling.

Taken together, these *in situ* data establish that AMD1 undergoes a biphasic response after AKI, characterized by an early induction in KIM-1+ injured proximal tubules followed by late-stage downregulation. Furthermore, this reduced tubular AMD1 is coupled to diminished proliferative activity and enhanced fibrotic progression, implicating AMD1-linked polyamine metabolism as a key metabolic feature of maladaptive repair.

### 3.3. Tubular AMD1 deficiency drives cell-cycle arrest and senescence, leading to maladaptive repair after AKI

**Figure 3.**
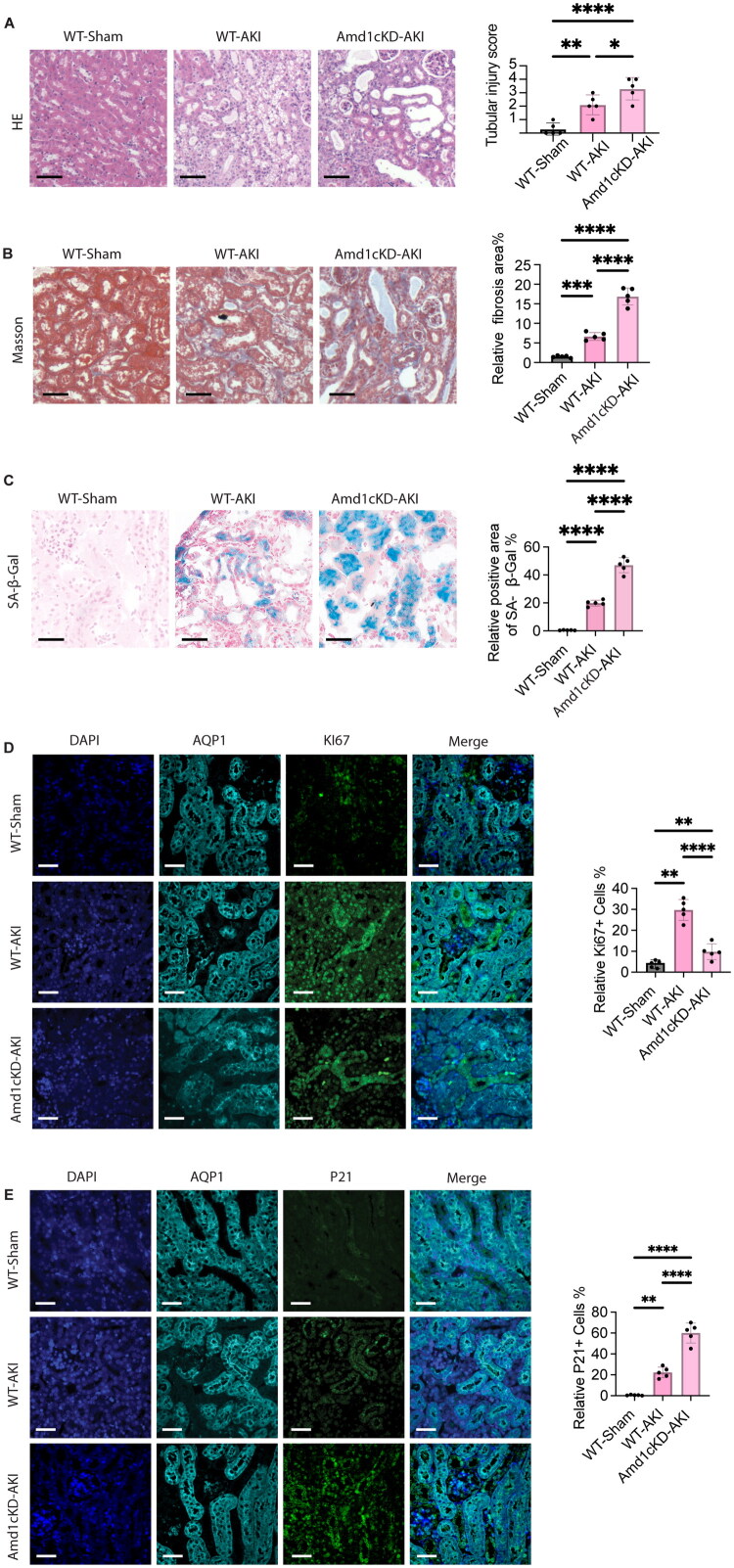
Tubule-specific knockdown of AMD1 exacerbates post-AKI injury and promotes tubular senescence. (A, B) Representative H&E staining (A) and Masson’s trichrome staining (B) showing renal histopathology and interstitial collagen deposition, respectively. The right panels show the quantification of the tubular injury score and relative fibrotic area. (C) Representative SA-β-Gal staining of kidney sections showing senescence burden; *Amd1* ^cKD^ mice exhibit a marked increase in SA-β-Gal-positive areas following AKI. (D, E) Representative immunofluorescence staining and quantification of the proliferation marker Ki67 (D, green) and the cell-cycle arrest marker p21 (E, green) within AQP1+ proximal tubules (cyan). Nuclei were counterstained with DAPI (blue). To ensure rigorous quantification and exclude injury-induced tubular autofluorescence, only Ki67+ and p21+ signals exhibiting strict nuclear co-localization with DAPI were included in the analyses. Scale bars = 50 μm. *n* = 5 per group. Data are presented as mean ± SEM. Statistical significance was determined using one-way ANOVA followed by Tukey’s *post hoc* test. *p < 0.05, **p < 0.01, ***p < 0.001.

To directly determine whether tubular AMD1 is functionally required for post-AKI repair, we subjected tubule specific Amd1 conditional knockdown mice (*Amd1 ^cKD^*) and littermate wild type (WT) controls to renal IRI induced AKI and evaluated repair outcomes at the late phase. IF and western blot results validated the Amd1 knockdown efficiency in *Amd1 ^cKD^* mice compared with WT mice (Figure S1B,C). Histological assessment by H&E staining revealed that, compared with WT-AKI kidneys, *Amd1 ^cKD^*-AKI mice displayed more severe tubular damage, accompanied by a significantly higher tubular injury score (Figure 3A). Consistent with these findings, Masson’s trichrome staining demonstrated that interstitial collagen deposition was markedly increased in *Amd1* ^cKD^ kidneys, with quantitative analysis confirming a significant elevation in relative fibrosis area compared with WT-AKI controls (Figure 3B).

Given that persistent senescence is a hallmark of maladaptive repair, we next examined senescence burden using Senescence-Associated β-galactosidase (SA-β-Gal) staining. While senescence was induced after AKI in WT kidneys, *Amd1* ^cKD^ mice exhibited a striking exacerbation of SA-β-Gal positivity, indicating a substantial expansion of senescent tubular areas (Figure 3C).

We then asked whether this repair failure was associated with impaired proliferative regeneration and activation of cell-cycle arrest pathways. Multiplex immunofluorescence staining in AQP1+ proximal tubules showed that Ki67+ proliferating tubular cells increased in WT kidneys after AKI, reflecting a regenerative response; however, this proliferative response was significantly blunted in *Amd1* ^cKD^ kidneys, as evidenced by a marked reduction in the proportion of Ki67+ cells (Figure 3D). In parallel, immunostaining for the cyclin-dependent kinase inhibitor p21 revealed that p21 expression was robustly upregulated after AKI and further intensified in *Amd1* ^cKD^ kidneys, supporting enhanced cell-cycle arrest in the absence of AMD1 (Figure 3E).

Collectively, these results demonstrate that deficiency of tubular AMD1 shifts the post-AKI trajectory toward persistent p21-associated cell-cycle arrest and heightened senescence, thereby suppressing tubular proliferative repair and promoting fibrotic remodeling, a phenotype consistent with maladaptive repair during the AKI-to-CKD transition.

### 3.4. Exogenous spermidine supplementation markedly alleviates AMD1 deficiency induced renal injury, senescence, and interstitial fibrosis

**Figure 4.**
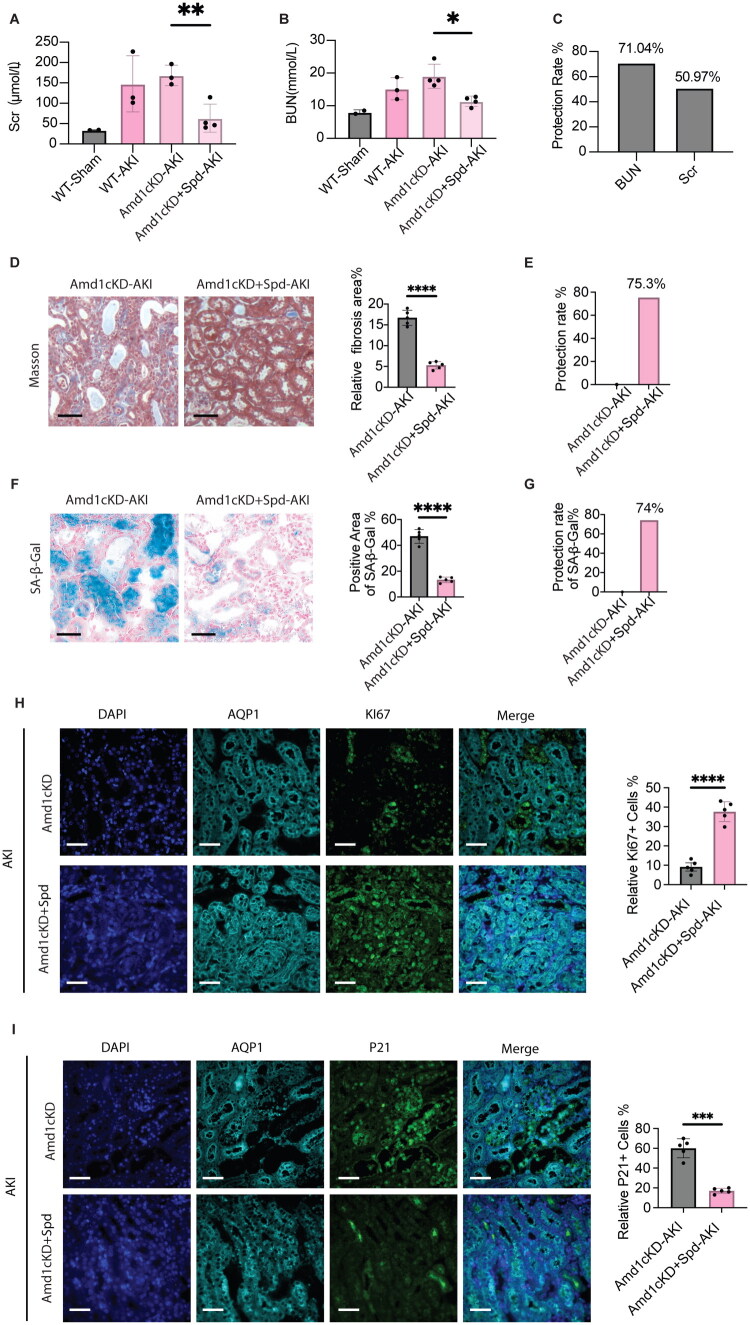
Exogenous spermidine supplementation markedly alleviates AMD1 deficiency-induced renal dysfunction, fibrosis, and tubular senescence. (A, B) Serum creatinine (Scr) (A) and blood urea nitrogen (BUN) (B) levels across the indicated groups. (C) Calculated renal functional protection rates conferred by spermidine (Spd) treatment for BUN and Scr. (D–G) Representative Masson’s trichrome staining (D) and SA-β-Gal staining (F) showing that Spd markedly reduces interstitial fibrosis and cellular senescence in *Amd1* ^cKD^ mice after AKI; the corresponding quantitative analyses and protection rates are shown in (E) and (G), respectively. (H, I) Representative multiplex immunofluorescence staining and quantification showing that Spd treatment increases the proportion of Ki67+ proliferating cells (H, green) and reduces p21+ expression (I, green) within AQP1+ proximal tubules (cyan). Nuclei were counterstained with DAPI (blue). Consistent with rigorous quantification standards, only Ki67+ and p21+ signals exhibiting strict nuclear co-localization were analyzed to exclude interference from tubular cast autofluorescence. Scale bars = 50 μm. *n* = 3–5 per group. Data are presented as mean ± SEM. Statistical significance was determined using one-way ANOVA followed by Tukey’s *post hoc* test (A, B) or two-tailed unpaired Student’s t-test (D, F, H, I). *p < 0.05, **p < 0.01, ***p < 0.001. (Note: For baseline comparisons corresponding to the WT-Sham and WT-AKI groups, please refer to Figure 3).

To determine whether reestablishing polyamine homeostasis could rescue the maladaptive phenotype caused by tubular AMD1 deficiency, we administered exogenous spermidine (Spd) to *Amd1* ^cKD^ mice after AKI. Serum biochemistry analyses showed that, compared with the *Amd1* ^cKD^ -AKI group, Spd significantly reduced serum creatinine (Scr) and blood urea nitrogen (BUN) (Figure 4A,B). When expressed as protection rates, Spd conferred 71.04% protection for BUN and 50.97% for Scr (Figure 4C), indicating a robust improvement in renal function despite AMD1 deficiency.

Histopathological evaluation further supported these functional benefits. Masson’s trichrome staining demonstrated that Spd treatment markedly decreased collagen deposition and interstitial fibrosis in *Amd1* ^cKD^ kidneys, reducing the fibrotic area from approximately 17% to 5% and yielding a 75.3% fibrosis protection rate (Figure 4D,E).

We next investigated whether Spd restored the imbalance between proliferation and senescence that characterizes maladaptive repair. SA-β-Gal staining revealed that Spd markedly reduced the senescent area in *Amd1* ^cKD^ kidneys, corresponding to an estimated 74% protection rate against senescence (Figure 4F,G). Consistently, immunofluorescence analyses showed that Spd suppressed the aberrantly activated ‘cell-cycle arrest-senescence’ axis in injured proximal tubules: p21 positivity decreased from 58% to 18%, whereas the proportion of Ki67+ proliferating tubular cells increased from 10% to 36% (Figure 4H,I).

Collectively, these results demonstrate that exogenous spermidine effectively corrects AMD1 deficiency induced polyamine depletion, restrains tubular senescence, re-enables cell-cycle reentry, and thereby suppresses chronic fibrotic remodeling while promoting functional renal repair after AKI.

### 3.5. AMD1 deficiency activates the DNA damage-p53/p21 axis and induces SASP programs, which are reversed by spermidine

**Figure 5.**
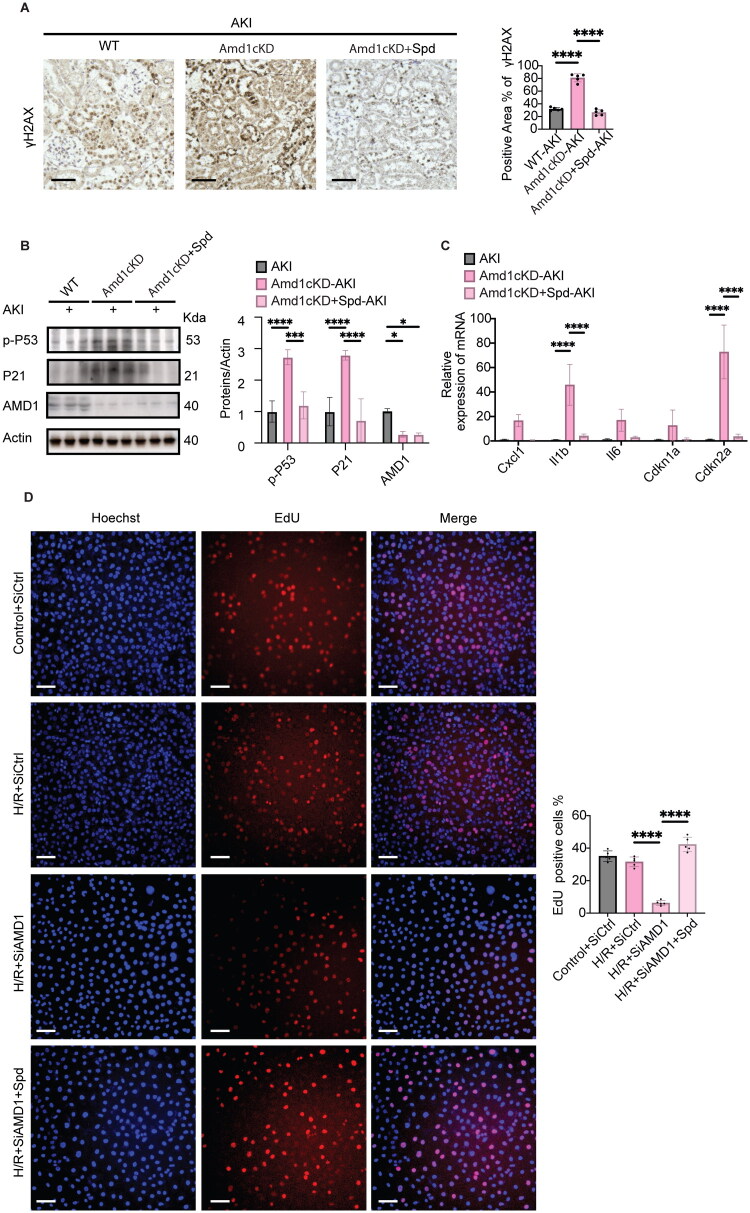
Tubular AMD1 deficiency exacerbates DNA damage accumulation and p53/p21-mediated proliferative arrest, which is reversed by spermidine. (A) Representative immunohistochemistry images and quantification showing elevated levels of the DNA damage marker γH2AX in kidneys from *Amd1* ^cKD^ mice following AKI, which is markedly reversed by exogenous spermidine (Spd) supplementation. (B) Western blot analysis and corresponding densitometric quantification of phosphorylated p53 (p-p53), p21, and AMD1 protein levels in kidney tissues across the indicated groups. β-Actin was used as the loading control. Blots are shown as representative cropped panels; uncropped full-length blots are provided in Supplementary Figure S3. (C) RT-qPCR analysis of senescence-associated secretory phenotype (SASP) factors (*Cxcl1*, *Il1b*, *Il6*, *Cdkn1a*, and *Cdkn2a*) in kidney tissues. (D)Representative images and quantification of EdU incorporation in HK-2 cells subjected to hypoxia/reoxygenation (H/R). Nuclei were counterstained with Hoechst (blue). The data demonstrate that AMD1 knockdown severely impairs DNA synthesis (EdU+, red), whereas Spd treatment effectively restores tubular proliferative capacity. Scale bars = 50 μm. *n* = 3–5 per group. Data are presented as mean ± SEM. Statistical significance was determined using one-way ANOVA followed by Tukey’s *post hoc* test. *p < 0.05, **p < 0.01, ***p < 0.001.

To elucidate the mechanism by which AMD1 deficiency drives tubular senescence, we focused on the maintenance of genomic stability. Higher polyamines, such as spermidine and spermine, act as essential organic polycations that directly bind to and stabilize chromatin, protecting DNA from oxidative stress and structural disruption [10,14]. Because AMD1 is critically required for the biosynthesis of these polyamines, its downregulation leads to intracellular polyamine depletion, which compromises genome integrity and promotes DNA damage [15,16]. Unresolved DNA damage is a well-established initiator of the senescence program. Therefore, we examined whether AMD1 deficiency drives tubular senescence through the activation of the DNA damage response and the subsequent p53/p21 checkpoint pathway. Immunohistochemistry for phosphorylated H2A histone family member X (γH2AX) revealed that, compared with WT kidneys, *Amd1* ^cKD^ kidneys displayed a significant increase in γH2AX-positive areas, indicating enhanced DNA damage accumulation and genomic instability upon AMD1 deficiency (Figure 5A). Western blotting further confirmed that DNA damage was accompanied by activation of the p53/p21 axis, as evidenced by increased levels of phosphorylated p53 (p-p53) and p21 in *Amd1* ^cKD^ kidneys (Figure 5B). Importantly, Spd supplementation markedly reduced γH2AX positivity and suppressed p-p53 and p21 induction (Figure 5A,B), suggesting that restoring polyamine availability mitigates DNA damage-driven checkpoint activation.

Because senescence is tightly linked to a pro-inflammatory secretory phenotype, we next assessed SASP-related transcripts. RT-qPCR analyses showed that *Amd1* ^cKD^ kidneys exhibited elevated expression of *Cxcl1, Il1b, Il6, Cdkn1a*, and *Cdkn2a*, consistent with amplification of senescence-associated inflammation, whereas Spd treatment broadly attenuated these SASP components (Figure 5C).

Then we validated the impact of this metabolic axis on proliferative capacity *in vitro* using a human proximal tubular cell line HK-2 hypoxia/reoxygenation (H/R) model. AMD1 knockdown severely impaired DNA synthesis, reducing the EdU-positive fraction to approximately 6.5%, while Spd supplementation restored EdU incorporation to 42.8% (Figure 5D). Furthermore, our *in vitro* targeted rescue experiments confirmed that exogenous spermidine, but not SAM, robustly suppressed AMD1 knockdown-induced DNA damage and p53/p21 checkpoint activation (Supplementary Figure S2A), solidifying that the phenotypes are primarily driven by polyamine depletion rather than one-carbon metabolic perturbations.

Together, these findings indicate that AMD1 preserves tubular proliferative competence by maintaining genome integrity and restraining DNA damage-p53/p21 checkpoint activation, thereby preventing the establishment of a self-reinforcing loop of DNA damage, senescence, and inflammation. Restoration of polyamine levels by spermidine interrupts this cascade and promotes adaptive repair following AKI.

## 4. Discussion

Previous studies on AMD1 have largely focused on its role in one-carbon metabolism, suggesting that AMD1 may influence DNA methylation and epigenetic status by altering the balance between S-adenosylmethionine (SAM) and decarboxylated SAM (dcSAM) [9,17]. Here, we emphasize AMD1 as a rate-limiting enzyme in polyamine biosynthesis and examine its function in the context of A-to-C repair failure, showing that AMD1-linked polyamine homeostasis is associated with tubular repair outcomes. In this study, proximal tubule-specific Amd1 conditional knockdown provides genetic evidence, and spermidine supplementation provides a pharmacological rescue, supporting that polyamine deficiency contributes to repair failure and that this phenotype is modifiable. Finally, most studies on polyamine metabolism in kidney diseases are descriptive, reporting changes in polyamine levels or the expression of polyamine-related enzymes and correlating these changes with pathological indices [18–20]. Causal evidence remains limited, as relatively few studies have tested polyamine pathways through genetic perturbation of key enzymes or direct manipulation of polyamine availability [21]. Beyond reporting metabolic alterations and phenotypes, we delineate a mechanistic link using evidence of γH2AX, p-p53/p21, SA-β-gal, and SASP-related transcripts, indicating that polyamine deficiency is associated with increased DNA damage and an enhanced DNA damage response, which in turn activates the p53/p21 checkpoint and promotes a sustained senescent state in tubular cells, thereby contributing to fibrotic progression.

Clinical and population studies suggest that changes in Spd levels are associated with kidney injury and its outcomes. In the population undergoing cardiac surgery, urinary Spd can be used to predict the occurrence of AKI during hospitalization and is associated with adverse outcomes, indicating that Spd may reflect early injury stress or differences in repair capacity [22]. In CKD cohorts, the serum polyamine profile is also disrupted, and Spd/spermine levels are correlated with eGFR and clinical outcomes, suggesting that systemic polyamine availability is related to disease severity [19]. At the level of human renal tissue and animal models, studies have also linked Spd or related axes to the process of renal fibrosis and shown that Spd supplementation can reduce fibrosis signals in specific models [23]. These pieces of evidence are mostly correlative or model-based support and are difficult to answer the questions of whether Spd changes are injury markers or pathogenic factors and which upstream link is more effective to intervene. Through conditional knockdown of Amd1 in proximal tubules, we demonstrated that the imbalance of polyamine homeostasis mediated by the upstream key enzyme AMD1 can promote the accumulation of DNA damage and activate the DNA damage response and p53/p21 checkpoint, maintaining tubular cells in a senescent and SASP state, thereby promoting fibrosis progression; while Spd supplementation can improve adverse outcomes at multiple readout levels, suggesting that restoring polyamine availability has intervention value when Spd metabolism insufficiency becomes a limiting factor. In summary, this study and previous evidence suggest that Spd can be used as a candidate metabolic marker for assessing the risk of kidney injury; meanwhile, the AMD1-Spd axis can be a candidate targeted pathway for subsequent clinical validation and intervention studies.

In the A-to-C process, we observed that AMD1 expression in proximal tubules was low at baseline, increased transiently in KIM-1+ injured tubules during the acute phase, and declined to baseline levels during the late repair phase. It is noteworthy that although late-phase AMD1 expression seemingly returns to its physiological baseline, the robust and persistent cellular stress during maladaptive repair imposes a heightened demand for polyamines to maintain genomic stability and support cellular proliferation. Therefore, this ‘baseline’ expression effectively constitutes a state of relative metabolic insufficiency. The failure to sustain AMD1 induction restricts polyamine availability, leaving the tubules vulnerable to unresolved DNA damage and driving them toward p53/p21-mediated senescence. AMD1 levels were positively associated with the repair index and inversely associated with fibrosis and α-SMA. Early AKI is accompanied by inflammation and oxidative stress, which can enhance polyamine catabolism and alter polyamine distribution, increasing the demand for compensatory polyamine synthesis [6]. AMD1 is regulated by intracellular polyamine levels through a negative-feedback mechanism that suppresses AMD1 translation *via* a polyamine-responsive upstream open reading frame (uORF) in its mRNA [24]. In addition, AMD1 maturation and enzymatic activity can be influenced by polyamines (e.g., putrescine), which may contribute to the transient increase in AMD1 during acute injury [25]. As injury persists and metabolic stress increases, polyamine homeostasis may become progressively impaired, which may contribute to reduced AMD1 levels in the late phase. AMD1 also links polyamine synthesis to one-carbon metabolism. By consuming SAM and producing dcSAM, AMD1 can affect methyltransferase activity and thereby influence methylation capacity [26]. Under sustained stress, such changes may contribute to more stable transcriptional programs and may affect the expression of AMD1 itself or gene networks involved in repair and fibrosis [4]. This can occur through reduced chromatin stability and DNA repair capacity, increased DNA damage signaling, and activation of the p53/p21 checkpoint [10,27]. In contrast, exogenous spermidine supplementation can partially restore higher polyamine availability and improve outcomes, including reduced fibrosis and improved proliferative capacity, potentially by lowering oxidative stress, activating stress-response pathways such as Nrf2 [23], and suppressing the p53/p21 axis [27].

In the context of the tubule-specific knockdown of AMD1, we observed an increase in γH2AX, accompanied by p53 phosphorylation and p21 upregulation. This suggests that AMD1 deficiency exacerbates DNA damage and enhances checkpoint activation. Firstly, AMD1 links carbon/methyl metabolism with polyamine synthesis: AMD1 consumes SAM and generates dcSAM, which can inhibit methyltransferase reactions, thereby affecting DNA as well as histone methylation and chromatin state [17,26]. An imbalance between SAM and dcSAM under continuous damage stress could lead to transcriptional changes in genes related to DNA replication, repair, and stress responses, reducing repair efficiency and making damage more prone to accumulating and triggering p53/p21 [17,26]. Secondly, as a key enzyme in polyamine synthesis, the absence of AMD1 reduces the availability of high-level polyamine supplies, particularly those related to Spd/spermine pools. Polyamines, as positively charged small molecules, participate in chromatin compaction, fork stability, and various DNA repair processes involving protein-DNA binding and assembly [28,29]. They also influence oxidative stress and mitochondrial homeostasis [30]. Thus, in the absence of AMD1, factors such as decreased chromatin stability, increased replication pressure, and elevated ROS burden could collectively amplify the DNA damage response, promoting p53/p21-mediated cell cycle arrest, and linking this to the observed aging and SASP amplification. Through these two possible axes, AMD1 can directly or indirectly influence DNA homeostasis. To distinguish between these two mechanisms, our *in vitro* rescue experiments provide direct evidence that polyamine depletion is the dominant metabolic driver of this maladaptive phenotype. Specifically, exogenous spermidine, but not SAM, robustly mitigates AMD1 deficiency-induced DNA damage and p53/p21 checkpoint activation.

Our study observed a biphasic change in tubular AMD1 following IRI. However, we did not perform continuous quantitative measurements of spermidine, spermine, and putrescine in renal tissue blood or urine at the same time points, nor did we simultaneously assess AMD1 enzyme activity or the polyamine metabolic flux. Consequently, it remains unclear whether the downregulation of AMD1 precedes the decline in spermidine or whether the changes in spermidine represent an early event of failed repair or a late consequence of metabolic depletion. Future studies that simultaneously measure AMD1 protein levels and activity, as well as the polyamine profile, at multiple critical time points, and validate these temporal relationships through staged dosing, could help clarify the indicators to be monitored clinically and the optimal timing for intervention. Another limitation is that our current mechanistic evidence primarily focuses on γH2AX increases and p53 and p21 activation, but lacks direct validation of the sources and repair mechanisms of DNA damage. For example, the role of stress-induced oxidative damage signals and specific repair pathways in this context remains unclear. Future research that concentrates on this aspect could be interesting, such as further distinguishing whether spermidine’s beneficial effects on renal outcomes are primarily due to its reduction of oxidative stress, improvement of mitochondrial function, or direct influence on chromatin stability and DNA repair processes.

Finally, while our study demonstrates that exogenous spermidine supplementation effectively mitigates AMD1 deficiency-induced renal injury and senescence, we did not comprehensively dissect the precise molecular mechanisms underlying this pharmacological rescue. It is critical to acknowledge that spermidine is a pleiotropic molecule with broad biological functions beyond simply replenishing the depleted intracellular polyamine pool. For instance, spermidine is a well-established, potent inducer of autophagy, which promotes cellular renewal and extends longevity [8]. Furthermore, it has been shown to exert profound antioxidant and anti-inflammatory effects [15] and improve mitochondrial quality control [31]. Consequently, the robust protective phenotypes we observed, such as the suppression of the p53/p21 senescence checkpoint and the reduction of fibrosis, might not exclusively result from direct DNA damage mitigation *via* chromatin stabilization. It is highly plausible that spermidine-induced autophagic clearance of damaged organelles acts in synergy with DNA protection to facilitate adaptive tubular repair. Unraveling the exact proportional contribution of autophagy induction versus direct polyamine pool restoration in the injured kidney represents a highly valuable direction for our future extended research.

## Supplementary Material

Supplementary_Table_S3_Key resources.pdf

Supplementary info file RF .docx

## Data Availability

The datasets analyzed in this study are publicly available in the Gene Expression Omnibus (GEO) under accession numbers GSE165876. Additional data supporting the findings of this study are available from the corresponding author upon reasonable request.
