# Supplementary material for: AMD1-mediated polyamine metabolism governs tubular repair fate by restraining senescence after kidney injury
Source: Ren Fail. 2026 Jun 14;48(1):2680375. doi: 10.1080/0886022X.2026.2680375 (PMC13267046; doi:10.1080/0886022X.2026.2680375)
Supplement: Supplementary_Table_S3_Key resources.pdf [file IRNF_A_2680375_SM2346.pdf]

Key resources and reagents

Type	Resource and Species	Source / Vendor	Catalog number	Application / Use
Antibody	phospho-p53 Rabbit Antibody	proteintech	80195-1-RR	WB 1:2000
	p21 Waf1/Cip1 (F-5) Antibody	Santa Cruz biotechnology,Inc.	sc-6246	WB 1:2000, IF 1:200
	Anti-GAPDH Rabbit antibody	Abcam	ab181602	WB 1:500
	alpha-Smooth Muscle Actin (D4K9N) Rabbit Monoclonal Antibody	Cell Signaling Technology	19245	IF 1:200
	AQP1 Rabbit antibody	proteintech	20333-1-AP	IF 1:200
	AMD1 Rabbit antibody	Abcam	ab127576	IF 1:200, WB 1:1000
	KIM-1 Rabbit antibody	proteintech	83221-2-RR	IF 1:200
	Ki-67 Rabbit antibody	proteintech	27309-1-AP	IF 1:200
	Histone H2A.X(γH2A.X) Monoclonal Mouse antibody	proteintech	68888-1-Ig	IHC 1:200
	Goat Anti-Mouse IgM HRP-Conjugated	Bioss	bs-0368G	IHC 1:200, WB 1:5000
	Goat Anti-Rabbit IgG H&L HRP-Conjugated	Bioss	bs-0295G	IHC 1:200, WB 1:5000
	Beta tubulin Recombinant Rabbit mAb	Bioss	bsm-33034R	WB 1:2000
	beta Actin Recombinant Rabbit mAb	Bioss	bsm-63325R	WB 1:2000
**Cell line**	HK-2	Procell	CRL-2190 BCRC	Human proximal tubule cells
**Mice**	C57BL/6J	Shanghai SLAC Laboratory Animal Co., Ltd	Stock# 000664	Background strain
**Reagents**				
Viral vector	AAV9-KSP-shAmd1(Pool)/AAV9-KSP-shCtrl (NC)	Hanbio Biotechnology Co., Ltd. (Shanghai, China)	Custom-made	2 × 10^12 vg/mL in PBS/0.001% Pluronic F-68; pyelocalyceal injection
IHC reagent	DAB chromogenic kit	Servicebio (Wuhan, China)	G1212-200T	Used for HRP–DAB development in IHC
Histology reagent	H&E staining kit	Servicebio (Wuhan, China)	G1005	Hematoxylin–eosin staining of kidney sections
Histology reagent	Masson's trichrome staining kit	Servicebio (Wuhan, China)	G1006	Collagen/fibrosis staining of kidney sections
Western blot reagent	SDS-PAGE gel preparation kit	Servicebio (Wuhan, China)	G2003	Preparation of resolving and stacking gels for SDS-PAGE
Western blot reagent	Ultra-sensitive ECL chemiluminescence kit	Servicebio (Wuhan, China)	G2074	High-sensitivity HRP chemiluminescent detection for immunoblotting
Protein ladder	Pre-stained protein marker	Servicebio (Wuhan, China)	G2089、G2091	Molecular weight standard for SDS-PAGE/Western blot
TSA IF reagent	TSA Plus Fluorescent Triple Staining Kit	Servicebio (Wuhan, China)	G1236-50T	Used for Multiplex immunofluorescence
TSA IF reagent	Hoechst	Byotime（Shanghai, China）	C1017	Nuclear counterstaining for IF and TSA (1 μg/mL)
Fix reagent	4% Paraformaldehyde	Servicebio	G1101	Fixation of renal tissues for histopathology and immunofluorescence.
TSA IF reagent	Anti-fade mounting medium	Servicebio	G1401	Mounting of slides/coverslips for fluorescence microscopy to prevent photobleaching.
Blocking	BSA (Albumin from bovine serum)	Servicebio	GC305010	Blocking and antibody dilution for IF/IHC (1%–5% w/v)
Cell culture	DMEM/F12 medium	Gibco	11320033	Base medium for HK-2 cell culture
Cell culture	Fetal Bovine Serum (FBS)	Gibco	10099141	Supplement for cell culture (10% v/v)
Transfection reagent	LipoMaster 3000	Vazyme (Nanjing, China)	TL301-01	Used for siRNA transfection
RNA extraction reagent	TRIzol reagent	AG (Accurate Biology, Hunan, China)	AG2102	Isolation of total RNA from cells and kidney tissues
Reverse transcription kit	HiScript III RT SuperMix	Vazyme (Nanjing, China)	R323-01	Reverse transcription of total RNA to cDNA
qPCR reagent	ChamQ SYBR qPCR Master Mix	Vazyme (Nanjing, China)	Q712-02	Real-time quantitative PCR with SYBR Green
Senescence staining reagent	SA-β-Gal staining kit (pH 6.0)	Servicebio (Wuhan, China)	G1073	Senescence-associated β-galactosidase staining of kidney sections or cells
Urea Assay Kit	Blood urea nitrogen (BUN) measurement	Nanjing Jiancheng Bioengineering Institute(Nanjing, China)	C013-2-1	Used for animal Blood urea nitrogen (BUN) measurement
Creatinine (Cr) Assay kit (sarcosine oxidase)	Blood creatinine (Scr) measurement	Nanjing Jiancheng Bioengineering Institute(Nanjing, China)	C011-2-1	Used for animal Blood creatinine (Scr) measurement
EdU Kit	EdU Cell Proliferation Kit	Byotime(Shanghai, China）	C0071S	EdU incorporation assay for cell proliferation
Chemical compound	Spermidine	MedChemExpress (MCE)	HY-B1776	10 mg/kg per day (in vivo); 10 μM (in vitro)
Chemical compound	S-Adenosyl-L-methionine disulfate tosylate(SAM)	MedChemExpress (MCE)	HY-W017770	10 μM (in vitro)
Chemical compound	Pentobarbital sodium	Sigma-Aldrich	Y0002194	40 mg/kg (in vivo)
