# Supplementary material for: AMD1-mediated polyamine metabolism governs tubular repair fate by restraining senescence after kidney injury
Source: Ren Fail. 2026 Jun 14;48(1):2680375. doi: 10.1080/0886022X.2026.2680375 (PMC13267046; doi:10.1080/0886022X.2026.2680375)
Supplement: Supplementary info file RF .docx [file IRNF_A_2680375_SM2345.docx]

**AMD1-Mediated Polyamine Metabolism Governs Tubular Repair Fate by Restraining Senescence after Kidney Injury**

Baiwei Mao ^1,2,3#^, Zhihuang Zheng ^1,2,3#^, Wenxin Fu ^1,2,3#^, Guozhe Cheng ^1,2,3^, Lijun Wang^1,2^, Jinfang Bao^1,2^, Xiaohua Liu ^4,5^, Hongbin Zhan ^4,5^, Miao Pan ^4,5^*, Jun Liu ^1,2,3^*

^1^ Department of Nephrology, Shanghai General Hospital, Shanghai Jiaotong University School of Medicine, Shanghai 201600, China

^2^ Laboratory of Nephropathy, Translational Medicine Center, Shanghai General Hospital, Shanghai Jiaotong University School of Medicine, Shanghai 201620, China

^3^ Institute of Translational Medicine, Shanghai General Hospital, Shanghai Jiaotong University School of Medicine, Shanghai 201620, China

^4^ Department of Nephrology, Shanghai First People's Hospital Ningde Hospital, Ningde, Fujian 352101, China.

^5^ Department of Nephrology, Ningde Municipal Hospital of Ningde Normal University, Ningde, Fujian 352101, China.

# Authors contributed equally.

*Correspondence:

Prof. Dr. Jun Liu, MD/PhD; Email: [liujun-sgh@sjtu.edu.cn](mailto:liujun-sgh@sjtu.edu.cn); Department of Nephrology, Shanghai General Hospital, Shanghai Jiao Tong University School of Medicine, Xinsongjiang Road 650, Shanghai, 201620, China. Tel: 021-63240090.

Prof. Dr. Miao Pan, MD; Email: [dr_pan_nnuh@yeah.net](mailto:dr_pan_nnuh@yeah.net); Department of Nephrology, Ningde Municipal Hospital of Ningde Normal University, Ningde, Mindong East Road 13, Fujian 352101, China.

**Supplementary Methods**

### In Vitro Metabolic Rescue Experiments with Spermidine and SAM

To evaluate the specific roles of polyamine versus one-carbon metabolism, metabolic rescue experiments were performed in HK-2 cells following Amd1 siRNA transfection. Immediately after the transfection procedure, cells were treated with either Spermidine (Spd, MedChemExpress, Cat# HY-B1776, 10 μM) or S-adenosylmethionine (SAM, MedChemExpress, Cat# HY-W017770, 10 μM). These cells underwent a 48 hour pre-treatment phase with the respective metabolites under normoxic conditions. Subsequently, the cells were subjected to the hypoxia and reoxygenation (H/R) protocol as described in the main text. Importantly, to ensure a continuous metabolic supply and prevent potential depletion during stress, the 10 μM concentration of Spd or SAM was strictly maintained in the culture medium throughout both the hypoxia and reoxygenation phases. After the completion of the H/R cycle, the cells were harvested for downstream molecular analyses.

**Supplementary Figure Legends**


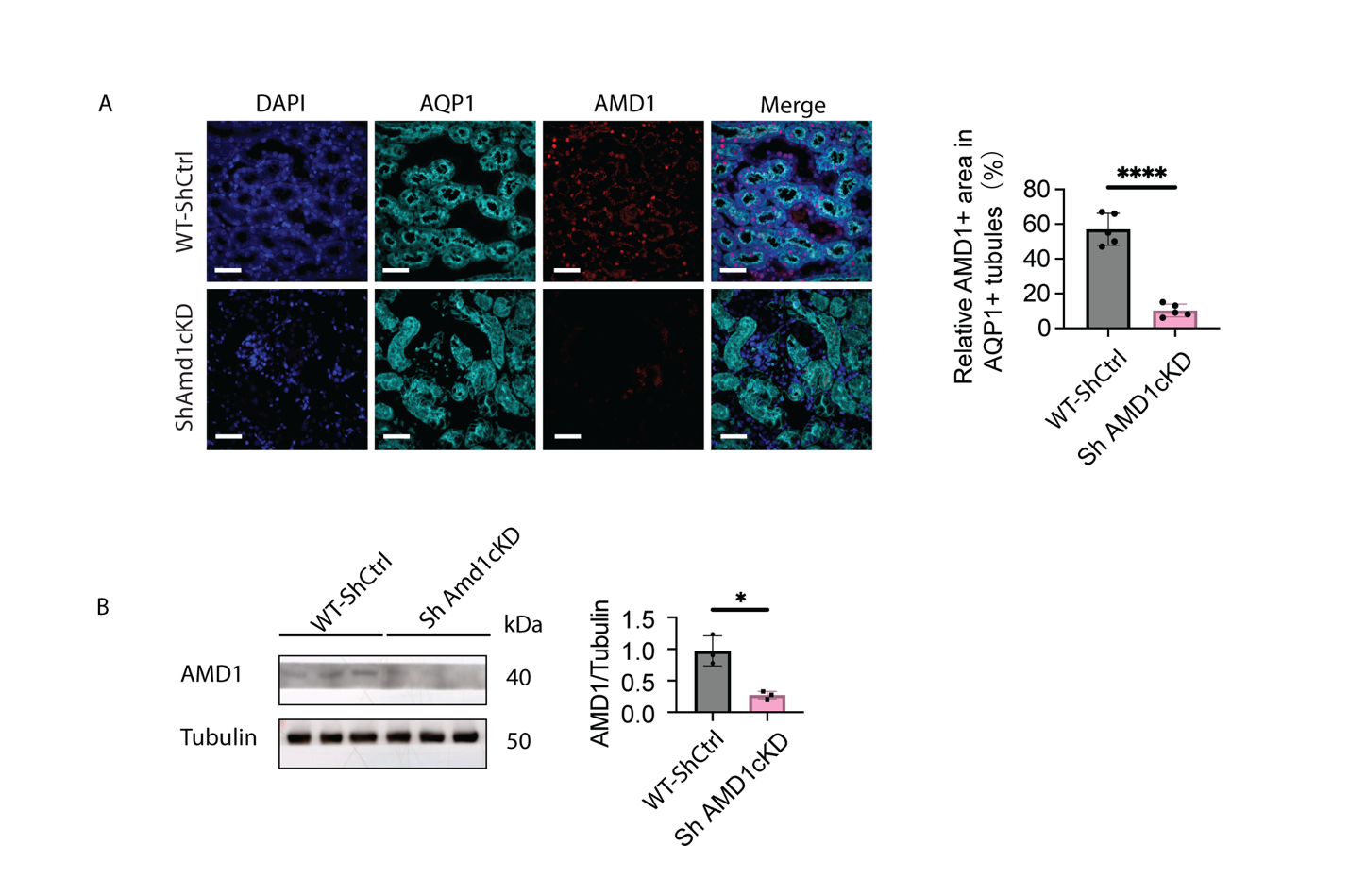


Supplementary Figure S1. Validation of tubule-specific *Amd1* knockdown efficiency. (A) Representative immunofluorescence co-staining and corresponding quantification demonstrating the robust, segment-specific knockdown of AMD1 protein (red) within AQP1+ proximal tubules (cyan) in *Amd1* cKD mice compared to wild-type (WT)-ShCtrl. Nuclei were counterstained with DAPI (blue). (B) Western blot analysis and densitometric quantification confirming the significant reduction of overall AMD1 protein levels in renal cortical tissues of *Amd1* ^cKD^ mice. Tubulin was utilized as the loading control. Uncropped full-length blots are provided in Supplementary Figure S4. Scale bars = 50 μm. Data are presented as mean ± SEM. Statistical significance was determined using a two-tailed unpaired Student's t-test. *P < 0.05, **P < 0.01, ***P < 0.001.

**
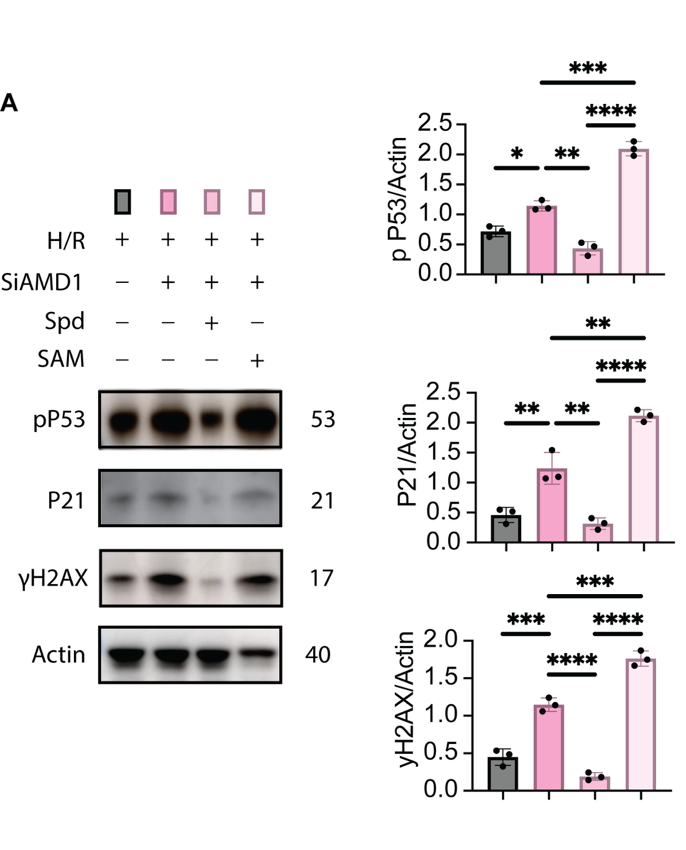
**

Supplementary Figure S2. Exogenous spermidine, but not SAM, robustly rescues AMD1 deficiency-induced DNA damage and p53/p21 checkpoint activation in vitro. (A) Western blot analysis and corresponding densitometric quantification of phosphorylated p53 (p-p53), p21, and γH2AX in HK-2 cells subjected to hypoxia/reoxygenation (H/R). Cells were treated with Amd1 siRNA (SiAMD1) and supplemented with either Spermidine (Spd, replenishing polyamine) or S-adenosylmethionine (SAM, replenishing one-carbon methyl donor). Actin was used as the loading control. Uncropped full-length blots are provided in Supplementary Figure S5. Data are presented as mean ± SEM (n = 3 per group). Statistical significance was determined using one-way ANOVA followed by Tukey’s post hoc test. *P < 0.05, **P < 0.01, ***P < 0.001, ****P < 0.0001.


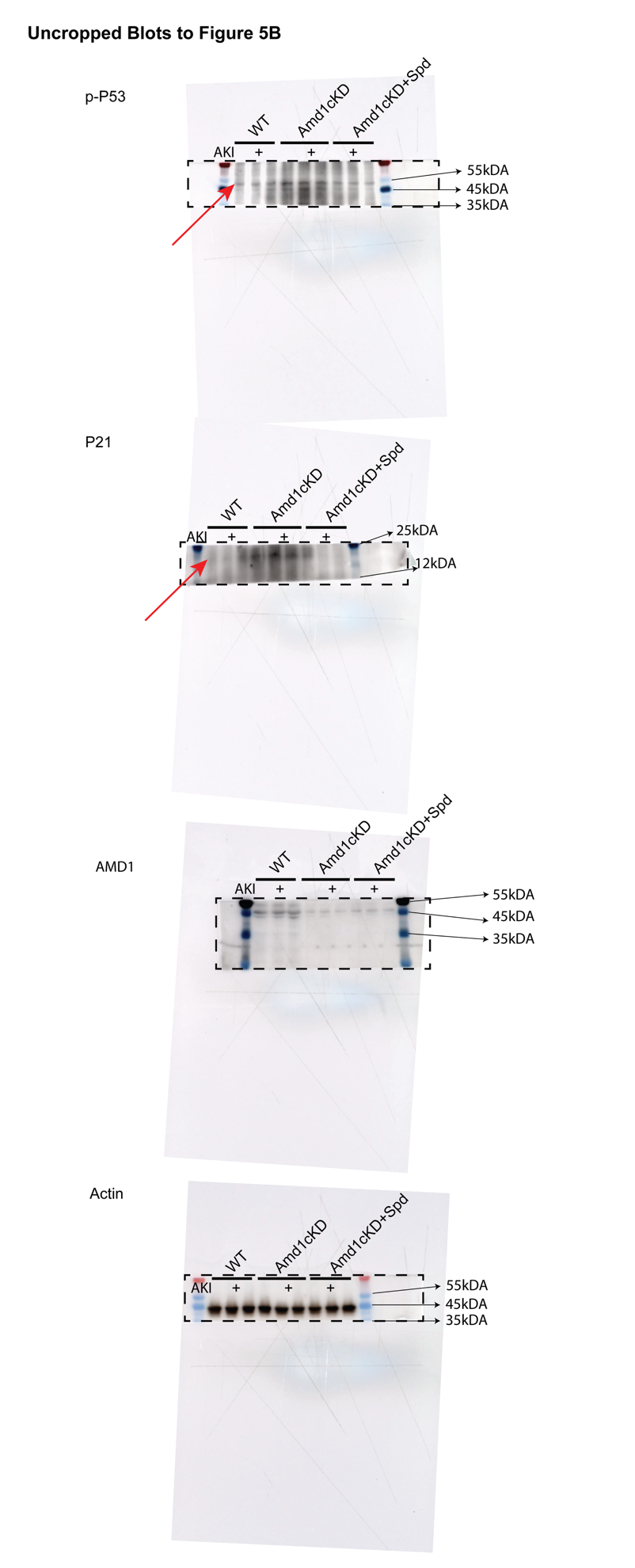


Supplementary Figure S3. Uncropped original Western blot images corresponding to Figure 5B. Full-length, unprocessed Western blot images for p-P53, p21, AMD1 and Actin (loading control) across the WT, *Amd1* cKD, and *Amd1*cKD+Spd groups following AKI (+). The black dashed boxes outline the specific cropped regions presented in the representative main text panels of Figure 5B. Red indicator arrows are specifically provided to explicitly identify the correct target bands utilized for densitometric quantification. The positions of corresponding molecular weight markers are indicated on the right.


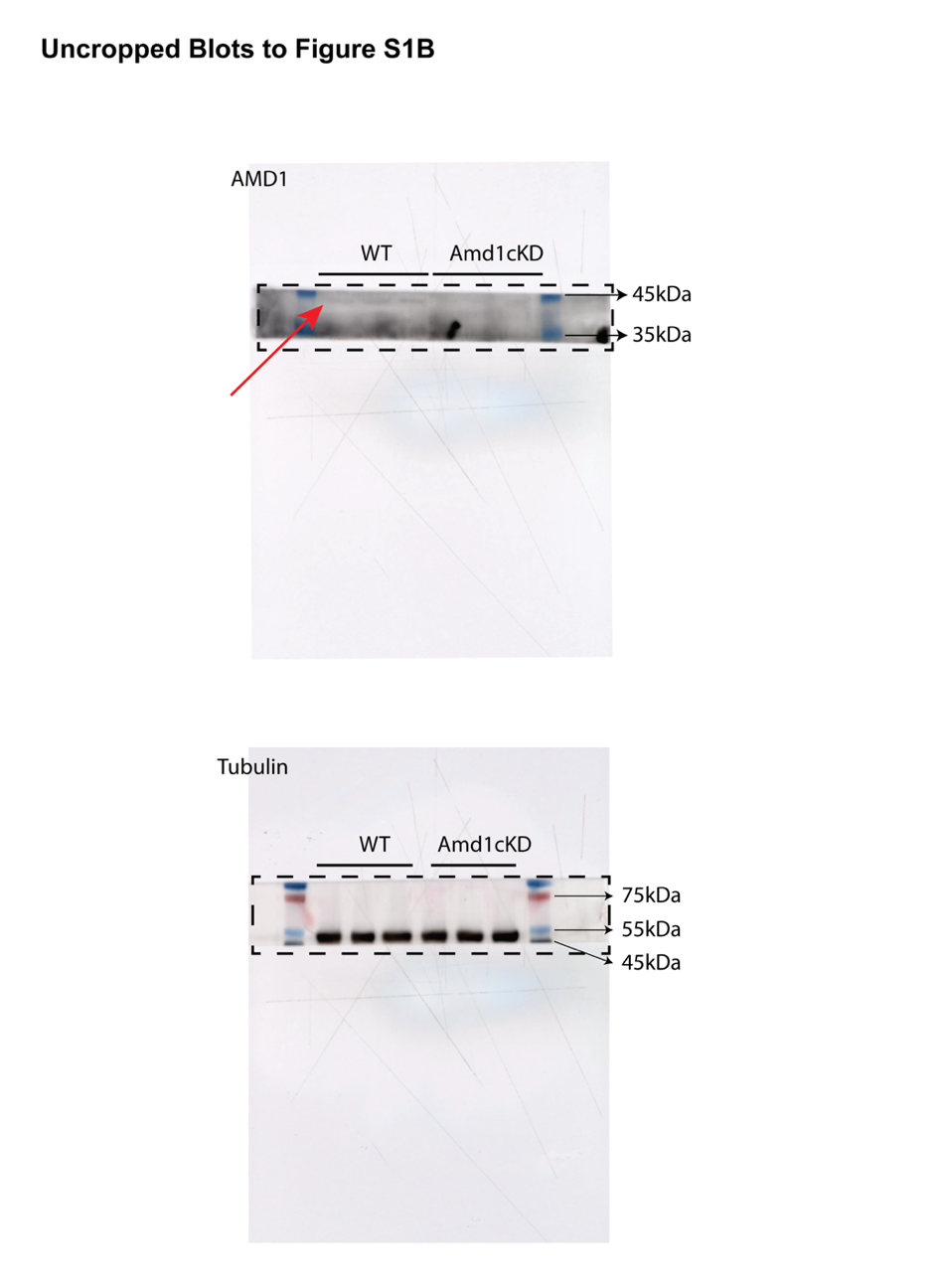


Supplementary Figure S4. Uncropped original Western blot images corresponding to Supplementary Figure S1B. Full-length, unprocessed Western blot images for AMD1 and Tubulin (loading control) across the wild-type (WT) and *Amd1* conditional knockdown (*Amd1* cKD) groups. The black dashed boxes outline the specific cropped regions presented in the representative panels of Supplementary Figure S1B. A red indicator arrow is specifically provided to explicitly identify the correct target band for AMD1 utilized to confirm the knockdown efficiency. The positions of corresponding molecular weight markers are indicated on the right.


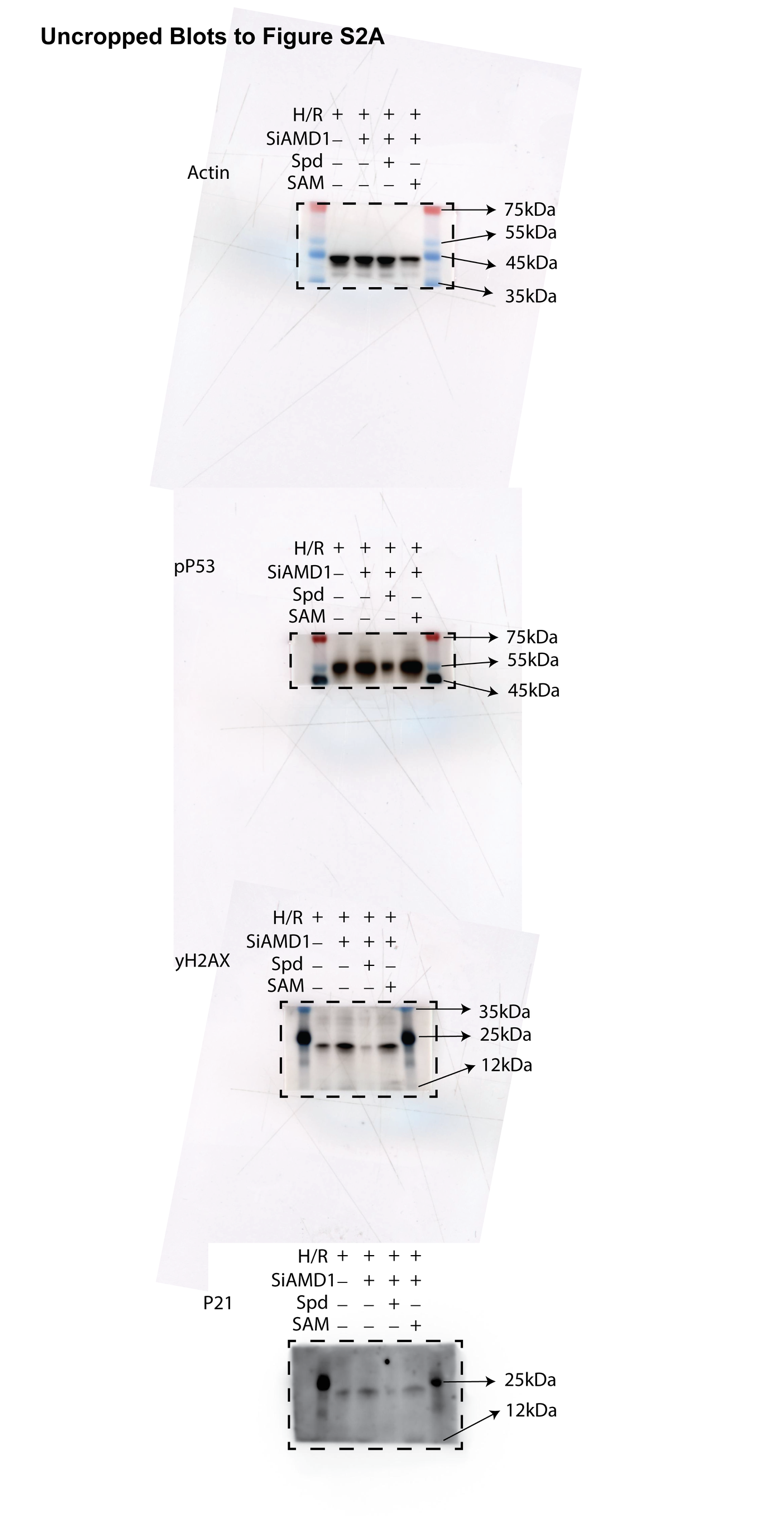


Supplementary Figure S5. Uncropped original Western blot images corresponding to Supplementary Figure S2A. Full-length Western blot images for Actin (loading control), p-p53, γH2AX, and p21 in HK-2 cells. Cells were subjected to hypoxia/reoxygenation (H/R) stress, treated with either negative control or *Amd1* siRNA (SiAMD1), and supplemented with Spermidine (Spd) or S-adenosylmethionine (SAM) as indicated. Black dashed boxes outline the specific cropped regions presented in the representative panels of Supplementary Figure S2A. The positions of the corresponding molecular weight markers are indicated on the right.

**Supplementary Table Legends**

Supplementary Table S1. Sequences of shRNAs.

| Target sequences designed for *Amd1* conditional knockdown. | | |
| --- | --- | --- |
| Construct Name | Target Region | Target Sequence（5'→3'） |
| AAV9-KSP-shAmd1 (Pool) | shRNA-1 | GCTTTCGGTCAGCTTTATC |
|  | shRNA-2 | CAGAGTCTACTTGGTTTGA |
|  | shRNA-3 | CTGTAGGTAAACTAGTGAT |
| AAV9-KSP-shCtrl | Scramble | TTCTCCGAACGTGTCACGT |

Supplementary Table S2. Sequences of siRNAs.

| SiRNA sequences for HK-2 | |
| --- | --- |
| Name | Sense Sequence (5' → 3') |
| Human AMD1-SiRNA1 | GGCGUUAUCAUUAUAUCUU |
| Human AMD1-SiRNA2 | GAACUUCAAAGAAUUCGAA |
| Human AMD1-SiRNA3 | UUCUAUGAAUGUCAUCAUU |
| Scrambled Control (SiNC) | UUCUCCGAACGUGUCACGU |

Supplementary Table S3 (Provided as a separate Excel file). Key reagents and primary antibodies used in this study. This table lists the primary antibodies, assay kits, and analytical reagents utilized in this study, including their commercial manufacturers, sources, and catalog numbers.

Supplementary Table S4. Sequences of qPCR Primers.

|  | Forward Sequence | Reverse Sequence |
| --- | --- | --- |
| *Cxcl1* | TCCAGAGCTTGAAGGTGTTGCC | AACCAAGGGAGCTTCAGGGTCA |
| *Il1b* | TGGACCTTCCAGGATGAGGACA | GTTCATCTCGGAGCCTGTAGTG |
| *Il6* | TACCACTTCACAAGTCGGAGGC | CTGCAAGTGCATCATCGTTGTTC |
| *Cdkn1a* | TCGCTGTCTTGCACTCTGGTGT | CCAATCTGCGCTTGGAGTGATAG |
| *Cdkn2a* | TGTTGAGGCTAGAGAGGATCTTG | CGAATCTGCACCGTAGTTGAGC |
